# Whole-blood transcriptomic findings in emergency department patients with suspected cannabinoid hyperemesis syndrome: a pilot study

**DOI:** 10.1186/s42238-026-00477-x

**Published:** 2026-07-23

**Authors:** Andrew Meltzer, Ziva D. Cooper, Ryan Heidish, Aditya Loganathan, Tarun Loganathan, Taylor Bolden, Isabella Lagunazad, John Perkins, Kevin Jaatinen, Jacob Adelman, Joseph Miller, Stephanie A. Eucker, Eileen Phillips, Timothy A. McCaffrey

**Affiliations:** 1 Department of Emergency Medicine, The George Washington University Medical Center, Washington, DC USA; 2 Center for Cannabis and Cannabinoids, Jane and Terry Semel Institute for Neuroscience and Human Behavior, Department of Psychiatry and Biobehavioral Sciences and Department of Anesthesiology and Perioperative Medicine, University of California Los Angeles David Geffen School of Medicine, Los Angeles, CA USA; 3 Department of Microbiology, Immunology, and Tropical Medicine and Department of Medicine, Division of Genomic Medicine, The George Washington University Medical Center, 2300 I Street NW, Ross Hall 205, Washington, DC 20037 USA; 4 Department of Emergency Medicine, Henry Ford Hospital, Detroit, MI USA; 5 Department of Emergency Medicine, Duke University Hospital, Durham, NC USA

**Keywords:** Cannabinoid Hyperemesis Syndrome, Cannabis, Cannabis allergy, Hypersensitivity, Transcriptomics, Blood biomarkers, Immune activation, HLA genotype

## Abstract

**Background:**

Cannabinoid hyperemesis syndrome (CHS) is a paradoxical and increasingly prevalent disorder characterized by recurrent vomiting in people with chronic cannabis use. Despite its growing clinical impact, the underlying mechanisms remain poorly understood.

**Methods:**

Genome-wide RNA sequencing was used to characterize transcriptomic differences and identify potential pathways involved in CHS pathogenesis. In this pilot study, whole blood RNA sequencing was performed on 7 patients with suspected CHS and 7 matched controls. Differentially expressed genes (DEGs) were identified, annotated and analyzed by automated and manual analysis. RNA sequences were further analyzed by digital isotyping for HLA Class I and II allele usage.

**Results:**

Suspected CHS was associated with marked activation of the adaptive immune system, including upregulation of B-cell related immunoglobin transcripts and altered expression of T cell, monocyte, and neutrophil-related transcripts. DEGs also suggested increased matrix degradation, and reduced adhesion and protease inhibitor transcripts. Digital HLA isotyping revealed increased MHC Class I expression, Class II allele restriction, and down-regulation of IgE receptor transcripts, a known response to elevated IgE levels in allergic hypersensitivity.

**Conclusions:**

Together, these preliminary findings identify immune-related signals in ED patients with suspected CHS, and lay the groundwork for future studies with larger, prospectively phenotyped cohorts to identify biomarkers, clarify immune triggers, and develop targeted therapies.

**Graphical Abstract:**

**Figure:**
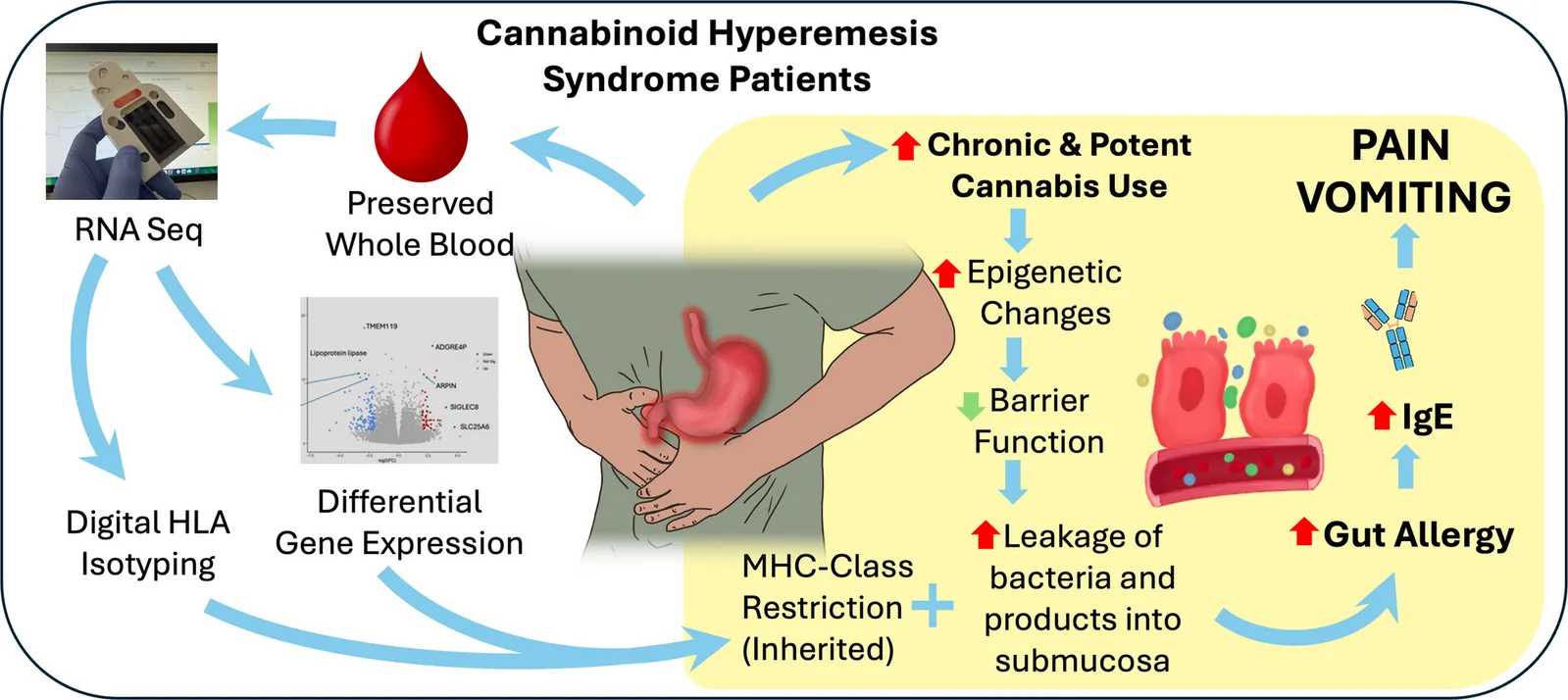

**Supplementary Information:**

The online version contains supplementary material available at https://doi.org/10.1186/s42238-026-00477-x.

## Key points

1. Central Problem: Cannabinoid hyperemesis syndrome (CHS) has become increasingly prevalent concurrent with both legalization and increasing potency of cannabis. Prior studies suggested there may be inherited changes in the endogenous cannabinoid system (ECS) that triggers a paradoxical hyperemesis in susceptible patients.
2. Key Results: There were not notable differences in the RNA levels of the cannabinoid receptors, but there were altered levels of eicosanoid pathway members that could impact endogenous cannabinoids. Notably, there were striking differences in the RNA expression levels of B cell immunoglobin genes, as well as higher expression of HLA Class I genes, and potential restriction of the HLA Class II allele usage in the suspected CHS patients. The pattern of changes signals the potential role of the adaptive immune response in CHS that may be more likely in subjects with specific HLA alleles.
3. Implications: Further studies should include analysis of the HLA allele usage and acquired immune pathways in CHS patients. Additional controls should include cannabis users without CHS, and patients with hyperemesis unrelated to CHS.

**Supplementary Information:**

The online version contains supplementary material available at https://doi.org/10.1186/s42238-026-00477-x.

## Background

Cannabinoid Hyperemesis Syndrome (CHS) is emerging as a critical public health issue, with the incidence of CHS growing by 150% annually over the past decade (Costales et al. 2024) and continuing to rise nationally due to the widespread legalization of cannabis alongside an increasing potency of cannabis products. As additional jurisdictions legalize cannabis use (Costales et al. 2024), there has been a 20-fold increase over the past 3 decades to about 17.7 million Americans that are daily or near-daily (DND) cannabis users (Caulkins 1979). Canada has experienced a similar increase in CHS incidence that tracks better with increased potency and physician awareness than with greater usage (Myran et al. 2022). This rise has coincided with a dramatic increase in the tetrahydrocannabinol (THC) content of marijuana, from less than 1% in the 1970 s to 4% in 1995 and 16% in 2022 (Cannabis 2024). Characterized by episodes of unremitting nausea, cyclic vomiting, and severe abdominal pain, CHS has become one of the most common specific clinical syndromes presenting to the emergency department (ED) in association with cannabis use (Monte et al. 2019; Shelton et al. 2020). CHS is characterized by recurrent episodes of severe nausea, abdominal pain, and painful vomiting, and can be associated with hot bathing behavior, which patients report can lessen symptoms (Venkatesan et al. 2014). In the absence of biomarkers, and having numerous possible etiologies, the diagnosis of CHS has been difficult, with an average range of 3–6 years before accurate diagnosis (Jiménez-Castillo et al. 2025).

CHS is paradoxical because cannabis is historically recognized for its anti-emetic properties, particularly for chemotherapy-induced nausea. Yet, in some chronic DND cannabis users, the drug becomes highly pro-emetic. Symptoms of CHS are minimally responsive to conventional anti-emetics such as ondansetron, but may respond to antipsychotics like haloperidol or olanzapine (Chopra et al. 2024). The most effective strategy for eliminating CHS and reducing repeat ED visits is cannabis abstinence (Hasler et al. 2025; Russo et al. 2022). Once affected, patients are prone to recurrence rapidly after resumption of cannabis use (Russo et al. 2022).

A prevailing theory suggests that CHS results from pharmacological overstimulation of the endocannabinoid system (ECS), especially through alterations in CB1 and CB2 receptor signaling (DeVuono and Parker 2020). The ECS is a complex network that regulates homeostasis throughout the body, including the gastrointestinal (GI) tract. Changes in the activity or expression of ECS enzymes and receptors have been proposed as one mechanism underlying CHS (DeVuono et al. 2024).

To better characterize the population affected by CHS, our group recently conducted a survey of 1,052 participants with self-identified CHS (Meltzer et al. 2025). Participants were typically young (mean age 32.1 years), initiated DND cannabis use at a mean age of 19.3 years, and reported high daily consumption (≥ 3 times daily, 82.2%). Rates of healthcare utilization were high, with 84.9% reporting at least one ED visit and 44.2% at least one hospitalization for CHS. Importantly, individuals who initiated DND cannabis use before age 18 were more likely to require hospitalization.

Despite the growing prevalence and burden of CHS, its underlying mechanisms remain poorly understood, and there are no established biomarkers to confirm diagnosis. The present study addresses this gap by applying genome-wide RNA sequencing to whole blood from CHS patients and matched controls. Our goals are to generate new hypotheses regarding CHS pathophysiology, identify non-invasive blood-based biomarkers, and lay the groundwork for future preventive and therapeutic strategies.

## Methods

### Patients

Patients with suspected CHS were enrolled in the Emergency Department (ED) and provided written informed consent under IRB Protocol #NCR 213728. Patients were diagnosed with suspected CHS using ROME IV criteria based on chronic cannabis use, recurrent stereotypical episodic nausea/vomiting with abdominal pain, supportive clinical features when available, and exclusion of alternative structural, surgical, infectious, or laboratory-defined causes during the ED evaluation. However, because it was impractical in the ED to ascertain symptom remission following sustained cannabis abstinence, the patients are considered as ‘suspected CHS (sCHS)’. These were not intended to represent first-time undifferentiated vomiting presentations, but rather patients with sCHS presenting for acute ED care. Control subjects were research or clinical staff matched for sex, and enrolled under a related IRB protocol #NCR213645 with explicit opt-in consent for future use of samples. Controls were not DND cannabis users nor reporting any vomiting. It was not appropriate or possible to involve patients or the public in the design, or conduct, or reporting, or dissemination plans of our research, although a large survey was conducted to understand CHS patients and their healthcare usage (Meltzer et al. 2025).

### RNA purification

Whole blood (6 ml) was collected into Tempus Blood RNA Tubes according to the manufacturer’s instructions and then frozen at –80 °C. High-quality mRNA was isolated from whole blood using the Invitrogen Tempus Spin RNA Isolation kit with an on-column DNase treatment to remove any contaminating genomic DNA. Final elution of RNA was performed using DEPC-treated water at 70 °C, yielding RNA that was stored at –80 °C until further use.

### RNA analysis and sequencing

Purified RNA was quantified using Agilent TapeStation analysis. A standardized amount of RNA from each sample was submitted to the GW Genomics Core Facility for sequencing, employing the Illumina Stranded Total RNA Preparation and Ligation with Ribo-Zero Plus, followed by a NextSeq 2000 P1 XLEAP-SBS 300 Cycle sequencing run. RNA and sequencing quality metrics are summarized in Table 1.

**Table 1:** Demographic and laboratory values of suspected CHS patients

	Patients		Controls
	Average	S.E.M	(or nml range)
N	7		7
Age (yrs)	28.60	3.2	42.71
Sex (%Male)	14.20		14.20
Race (%White)	0		57.10
Temp (C)	36.81	0.05	37
Systolic BP (mm Hg)	148.43	16.21	120—130
Haldol Rx (%)	100.00		0
White Blood Cell Ct (K/uL)	10.37	1.27	4.0—11.0
Red Blood Cell Ct (M/ul)	4.73	0.34	4.1—5.9
Hemoglobin (g/dL)	13.00	0.77	12.1—17.2
Hematocrit (% RBC)	38.26	1.95	36.1—50.3
Mean Corp Vol (fL)	81.90	2.67	80—100
Mean Corp Hemo (pg)	27.79	1.05	27—34
Mean Corp Hem Conc (g/dL)	33.93	0.81	32—36
RBC Dist Width-CV (%)	13.81	1.07	11.5—14.5
Platelet count (K/uL)	329.57	32.17	150—450
Mean Platelet Vol (fL)	10.24	0.22	7.5—12.0
Neut Ct (K/uL)	8.14	1.23	1.8—7.7
Lymph Ct (K/uL)	1.50	0.14	1—4.8
Neut/Lympho Ratio (NLR)	5.70	1.00	1.0—3.0
Mono Ct (K/uL)	0.66	0.12	0.2—0.8
Eos Ct (K/uL)	0.02	0.01	0.0—0.4
Baso # (K/uL)	0.02	0.01	0.0—0.1
Immature Gran/Bands (%)	0.04	0.01	0.0–0.05
RNA Yield (ug/3 ml)	17.84	5.26	11.47
RNA Integrity (RIN)	8.37	0.11	8.27
Total Paired Reads	17106641	707816	16703231

### RNAseq analysis

Paired-end FASTQ files were uploaded to the Galaxy platform and processed using standard settings. Low quality reads were removed with Trimmomatic, and sequence quality was measured using FastQC. Alignment was performed against the HG38 genome using HISAT2. Transcript counts were obtained with FeatureCount, and differential expression analysis was performed with DESeq2. Gene classification was conducted via automated ontology/pathway analysis using NIH DAVID (Sherman et al. 2021), supplemented by manual curation through PubMed and GeneCard searches.

### Blood microbiome ddPCR

To quantify total bacterial 16S and fungal 28S DNA fragments in blood, DNA was isolated from Tempus-preserved samples with minor modifications to published methods (Ferrante et al. 2018). Briefly, microbial lysis was achieved by treating samples with a lytic chemical solution and subjecting them to multiple freeze/thaw cycles, followed by nucleic acid precipitation using phenol/chloroform/isoamyl alcohol. Purified DNA (50 ng) was analyzed by ddPCR using primers specific for bacterial 16S (Gram-negative and Gram-positive) and fungal 23S rRNA, as previously described (Klaschik et al. 2002).

### HLA isotyping

Seq2HLA, a Python/R-scripted tool, was used to generate probable human leukocyte antigen (HLA) genotypes from RNA-Seq datasets. FASTQ files were aligned using Bowtie2 to a custom index of all known HLA alleles. Class I and II HLA expression was quantified, and HLA haplotypes were inferred to 4-digit resolution, accompanied by a confidence p-value for each call. Analyses were performed on the Galaxy platform using default parameters.

### ddPCR confirmation of host immune activation markers

Host immune RNA levels were quantified using the Bio-Rad Digital Droplet PCR (ddPCR) System. Extracted RNA was reverse transcribed into cDNA using gene-specific primers and RNAse H + reverse transcriptase (iScript, BioRad). cDNA was partitioned into droplets, PCR amplification was performed on a Bio-Rad C100 thermocycler, and probe fluorescence intensity was measured using the Bio-Rad QX200 Reader.

### Statistical analysis

Summary statistics for clinical and lab values between groups was computed and compared by t-test for continuous variables or chi-square for discrete variables. RNAseq analysis was analyzed by DeSeq2 which uses a negative binomial generalized linear model to compute fold change estimations and adjusted *p*-values for the false discovery rates (Love et al. 2014). Cell type comparisons were compared between groups by t test. Digital HLA isotyping was analyzed by comparing locus-specific HLA expression levels by t test, and comparing allele isotypes with non-parametric chi-square testing.

## Results

### Patient characteristics

In this pilot study, seven sCHS patients were enrolled for in-depth RNAseq analysis of whole blood RNA. Their demographic and clinical laboratory information is summarized in Table 1. Control subjects were recruited from the clinical/research staff with an effort to match gender, because six of the seven sCHS patients were female. Notable features among sCHS patients included a predominance of females (6/7), self-reported Black race (7/7), a borderline elevated white blood cell count (10.4 K/µL, where normal is 4–11 K/μL), an elevated neutrophil count (8.14 K/µL, where normal is 1.8–7.7), and an elevated neutrophil/lymphocyte ratio (5.7, where normal is 1–3.5). Control subjects were more racially diverse (4 White, 1 Black, 1 Asian, 1 Hispanic), and complete blood counts (CBCs) were not performed (normal reference ranges shown). Co-morbidities among the sCHS cohort included a hemorrhagic ovarian cyst (Costales et al. 2024), esophageal perforation with pneumomediastinum (Costales et al. 2024), and chronic gastritis with hypertension (Costales et al. 2024). All seven sCHS patients received haloperidol (Haldol) in the ED for CHS management.

### RNAseq analysis of whole blood RNA

RNA purification yielded somewhat, though not significantly, higher total RNA in sCHS patients compared to controls (CON = 11.5 μg/3 mL blood, sCHS = 17.8 μg/3 mL, *p* = 0.26), a difference largely attributed to a single sCHS patient with an unusually high RNA yield. Both groups demonstrated high and comparable RNA quality (RIN scale 1–10: CON = 8.27, sCHS = 8.37, *p* = 0.46) as assessed by Agilent TapeStation. Illumina RNAseq produced similar numbers of paired end reads for sCHS patients (17.1 million) and controls (16.7 million, *p* = 0.77). Aligned read counts were also comparable at about 50% of total reads between groups.

DeSeq2 analysis identified 220 transcripts upregulated and 140 transcripts downregulated (fold change > 4, *p* < 0.05 uncorrected; see Supplementary Data 1 for volcano plot). All transcript data, including raw read counts, normalized read counts (nRC), fold changes, and *p*-values for the 360 differentially expressed genes (DEGs) are provided in Supplementary Data 2. Transcripts were annotated by both automated and manual approaches and subsequently grouped into relevant classifications, such as upregulated (Table 2) and downregulated transcripts (Table 3).

**Table 2:**
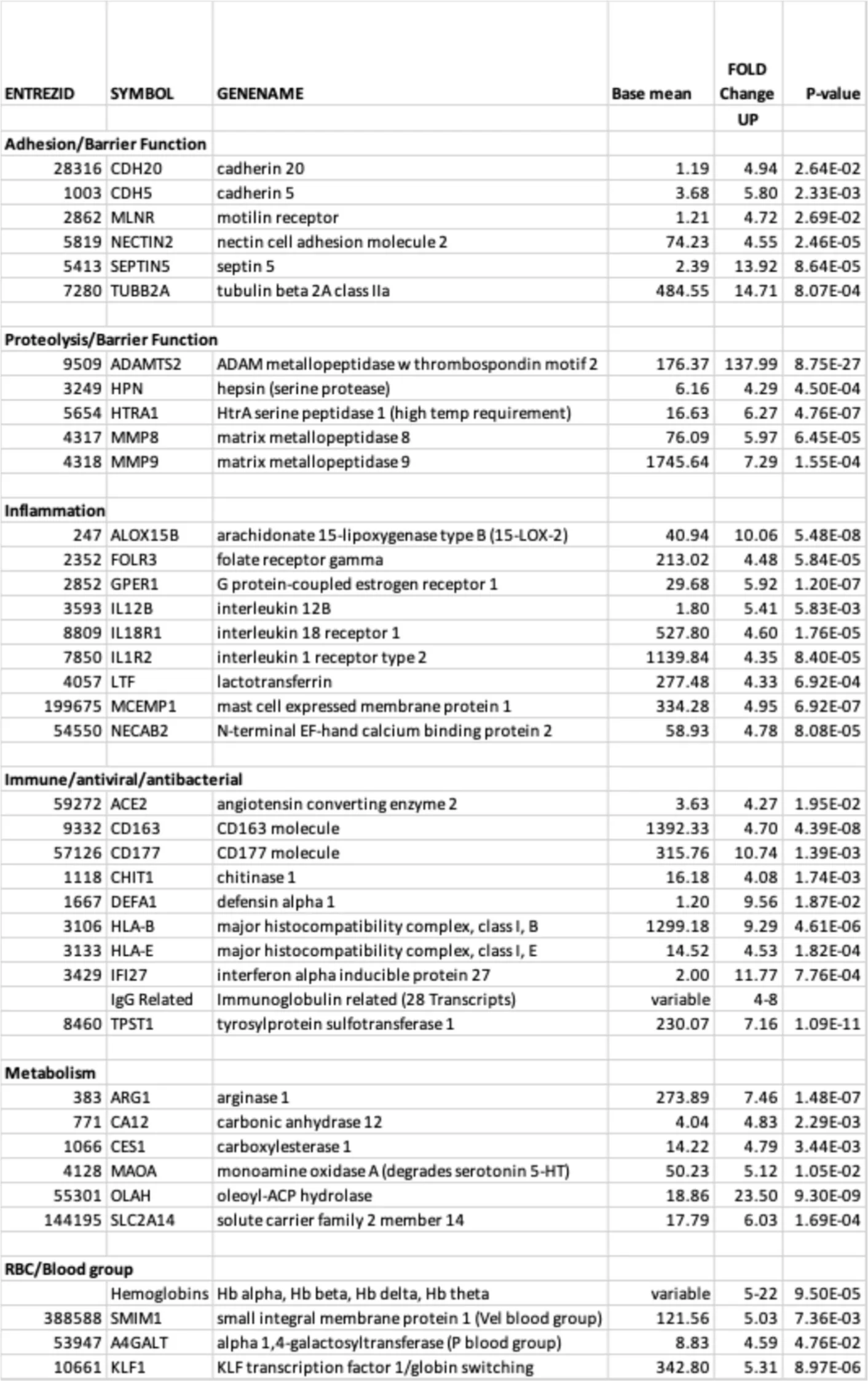
Differentially expressed genes increased in suspected CHS

**Table 3:**
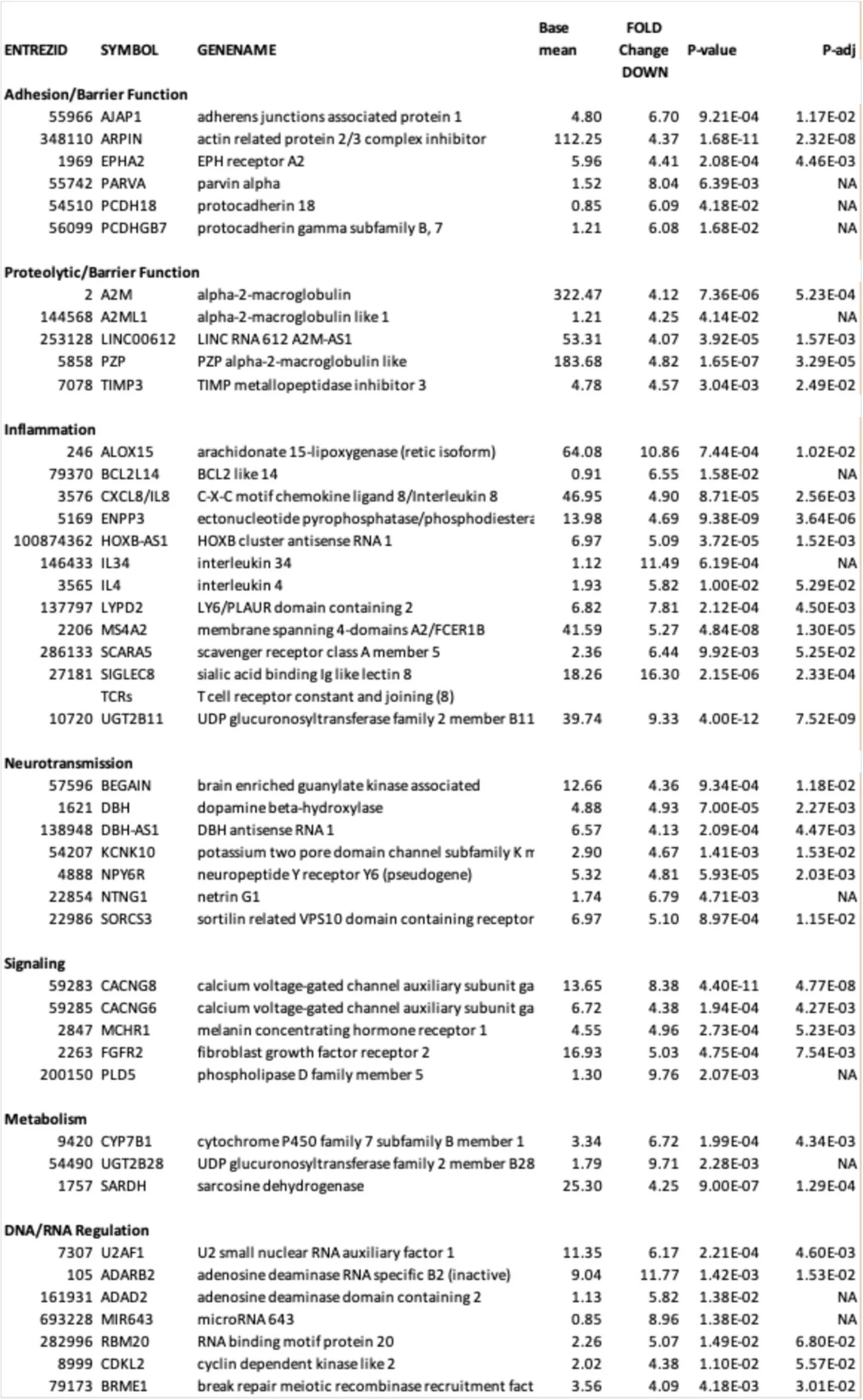
Differentially expressed genes decreased in suspected CHS

**Fig. 1:**
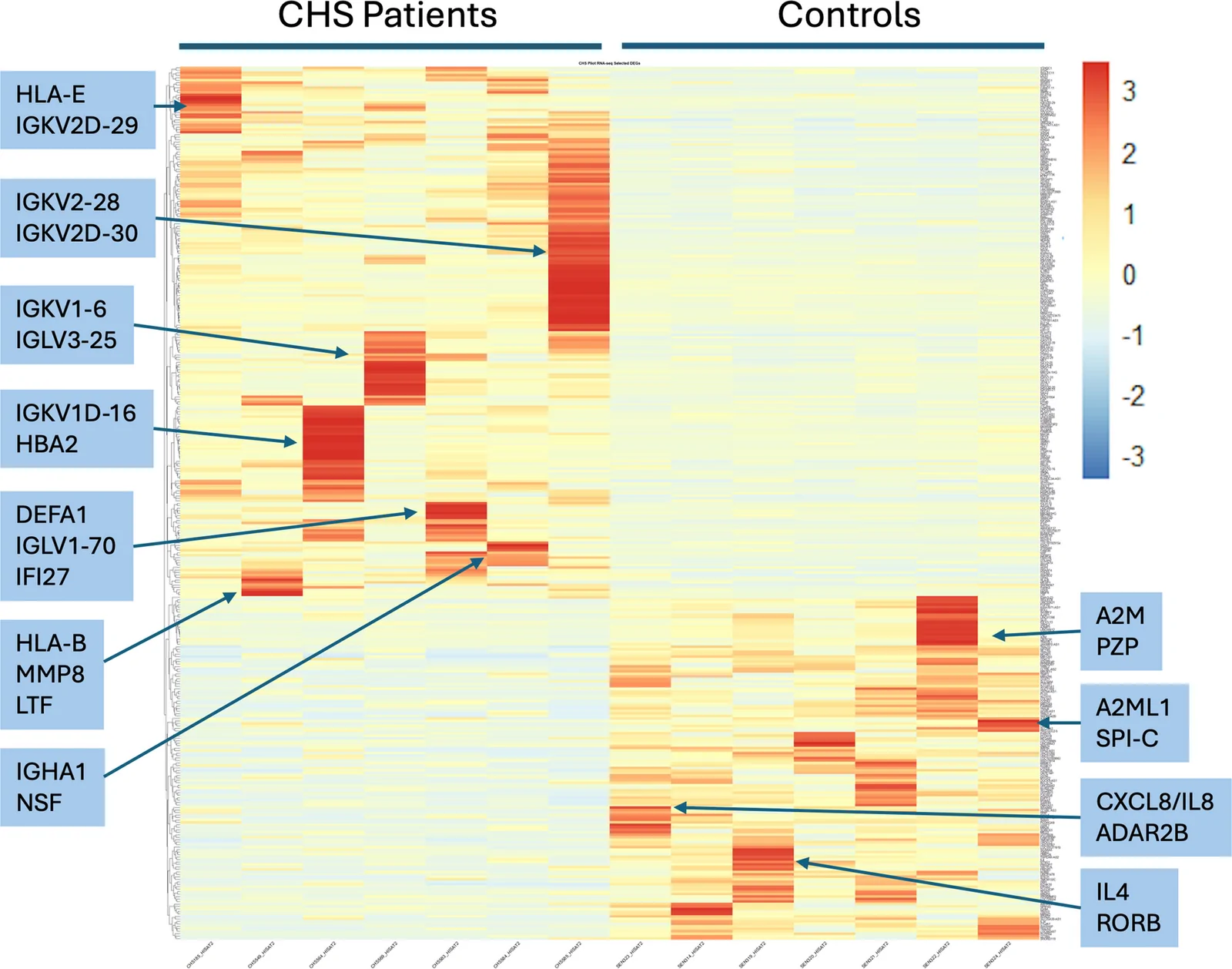
Hierarchical clustering of differentially expressed genes in whole blood of suspected CHS patients. DeSeq2 analysis identified 240 upregulated and 120 downregulated transcripts. Hierarchical clustering was used to identify patterns of covariance between transcripts across the seven sCHS patients and seven controls (X-axis). Changes in transcript levels are normalized per transcript and are color-coded on a log_2_ scale (dark red = 3log_2_ = 8 × increase; dark blue = –3log_2_ = 8 × decrease). Selected transcripts are highlighted

Hierarchical clustering of the 360 DEGs revealed that upregulated transcripts exhibit a unique pattern in each sCHS patient, as opposed to a uniform pattern across the cohort (Fig. 1). Examination of the most elevated transcripts in individual patients (“hot” clusters) showed each included some Ig-related transcripts (e.g., HLA-E and IGKV2D-29), indicating that while each patient mounts a distinct immune response, there is a shared elevation of immune activation markers. The larger number of increased versus decreased transcripts reflects individual variation among sCHS patients, primarily within the adaptive and innate immunity gene families.

### Cannabinoid pathway

To examine the potential role of cannabis-induced changes in the endocannabinoid system (ECS), the major gene transcripts associated with the ECS were examined. A prior small-scale study had identified some potential DNA variants in ECS-related genes associated with CHS (Russo et al. 2022). The expression levels of key ECS-associated transcripts as well as those previously linked to CHS (Russo et al. 2022), encompassing cannabinoid receptors (CNR1, CNR2), TRPVs (Costales et al. 2024; Caulkins 1979; Myran et al. 2022; Cannabis 2024; Monte et al. 2019; Shelton et al. 2020), transporters/metabolizers (Monte et al. 2019), and signaling (Myran et al. 2022) were compared between groups (Supplementary Data 3). Even with a reduced threshold of 2-fold change, none of these transcripts were significantly altered. A nearly twofold increase in dopamine receptor D2 (DRD2) was observed, but the absolute expression level was low (nRC = 0.16,*p* = 0.76), limiting confidence in this result. However, as shown below, there are notable changes in the eicosanoid pathway, which overlaps the ECS by virtue of shared use of arachidonate metabolites to create endogenous cannabinoids.

### Cell type analysis

Even on cursory examination, noticeable changes were observed in transcripts associated with specific cell types—especially B cells, T cells, monocytes, and neutrophils. This was objectively assessed using a two-pronged approach employing a blood cell transcript database (Blood Atlas) derived from single-cell and sorted cell type RNAseq studies (Uhlen et al. 2019). First, DEGs were mapped to known cell types by comparing their expression levels.

**Fig. 2:**
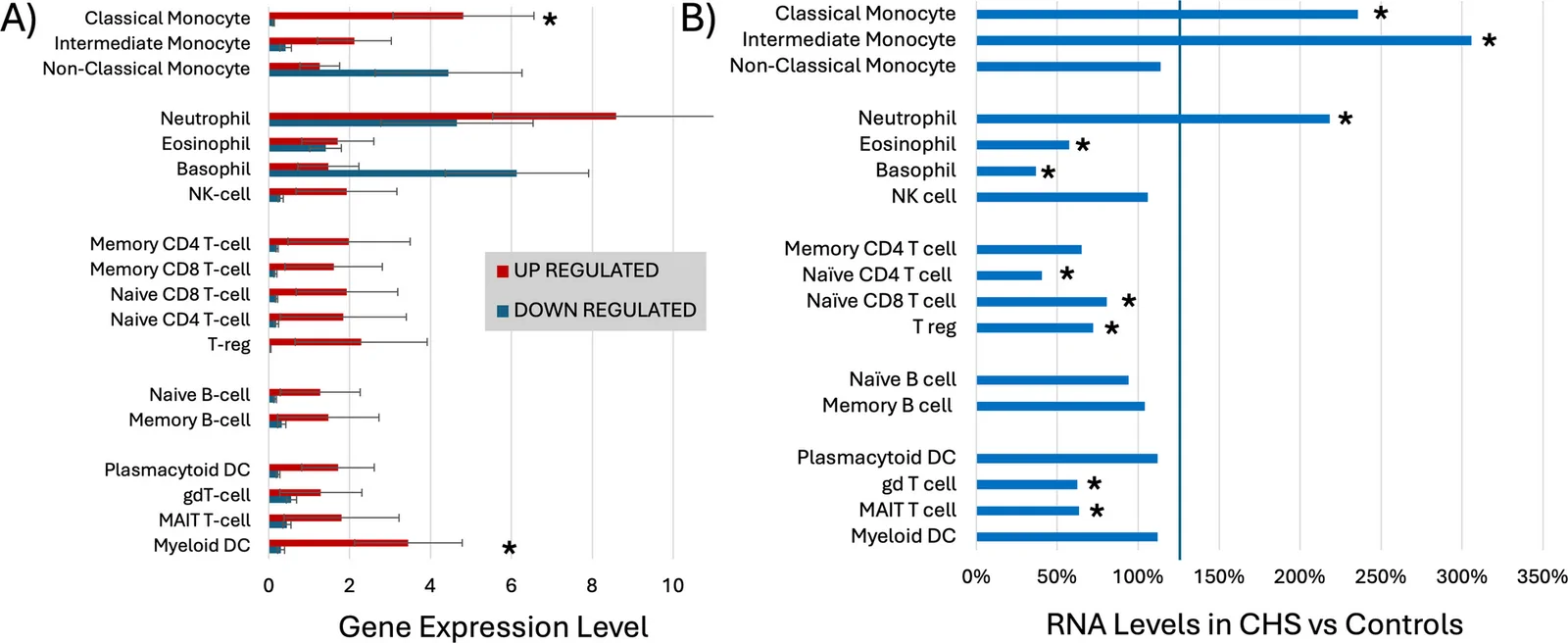
Cell type analysis in RNAseq of suspected CHS patients. **A** DEG expression across major blood cell types: Upregulated (red) and downregulated (blue) DEGs were mapped against the Blood Atlas, with normalized read counts (nRC) used to indicate DEG transcript abundance by cell type. **B** Cell-specific markers in sCHS RNAseq data: Pre-curated, cell type-enriched RNA transcripts were analyzed for expression across sCHS and control groups. The average expression in sCHS patients was expressed as a percent of control levels. *Asterisk indicates p* < *0.05 between groups*

Of the 360 DEGs, 63 could be mapped to the Blood Atlas. The average gene expression among the mapped upregulated versus downregulated transcripts is shown for their expression in the major cell types (Fig. 2A). Upregulated transcripts were most highly expressed in classical monocytes, neutrophils, and myeloid dendritic cells (DC). Conversely, downregulated transcripts were expressed mainly in non-classical monocytes, neutrophils, and basophils. Thus, while the DEGs were associated with particular cell types, they were also expressed across various immune cell populations.

As a second approach, pre-curated lists of cell type-enriched transcripts, unrelated to the DEGs, were averaged for each group to assess relative abundance changes. Average expression levels of curated cell-type markers showed that sCHS patients had increased levels of markers for classical/intermediate monocytes and neutrophils, but decreased markers for eosinophils, basophils, T cells, γᵟT-cells, and MAIT cells (Fig. 2B). Notably, RNA markers of B cells were not different between groups. Because these markers are independent of the DEGs, the data suggest potential shifts in immune cell populations, although changes in activation state could also account for the observed transcript fluctuations.

### B cell-related transcripts

Further manual analysis showed that among the increased transcripts, 28 transcripts were clearly related to B cell immunoglobulins, exhibiting 4–8-fold increases in sCHS patients (Table 2). B-cell activation has also been described in irritable bowel syndrome (IBS) (Ohman et al. 2009). However, Netrin G1 (NTNG1), another B-cell–related transcript, was reduced in sCHS patients.

### T cell-related transcripts

Eight downregulated transcripts were related to T cell receptors (TRAJs 8, 15, 30, 50; TRAV8-1; TRBC2; TRBJ1-4; TRDV2), with decreases of 4–6-fold in sCHS (Table 3). Additional T cell–specific changes were explored in the cell type analysis above.

### Innate immune cell-related transcripts

A subset of upregulated DEGs corresponded to markers of innate immune activation previously identified in studies of bacterial, viral, and biofilm infections in abdominal pain, pneumonia, or COVID-19 patients (Chawla et al. 2016; Meltzer et al. 2023; Wargodsky et al. 2022). Notable increases included DEFA1, ALPL, and IFI27, as well as neutrophil-related (DAAM2, VNN1) and monocyte/macrophage markers (SIGLEC11, VSIG4).

### Red blood cell-related changes

sCHS patients had marked increases in transcripts for hemoglobins HbA, HbB, HbD, and HbTheta with 5–22-fold elevations over controls (Table 2). Hb transcript changes are noted in spaceflight and bedrest (Trudel et al. 2024), as well as in high-altitude climbers (Chen et al. 2012). These changes may reflect physiological stress from emesis rather than intrinsic RBC abnormalities, as CHS patients have been reported to exhibit electrolyte disorders, hypertension, chronic lung disease, and anemia (Madireddy et al. 2019). Although racial differences and sickle cell carrier status could be relevant, the transcript elevations were consistent across patients, with no clinical or RNAseq evidence for sickle cell disease, although two were likely carriers (Gee et al. 2021).

### Allergic/immune changes

Three transcripts (HLA-B, HLA-E, HCP5) suggested a possible immune genotype associated with CHS. HLA-B genotypes and RNA levels have previously been linked to inflammatory disorders such as irritable bowel syndrome (IBS) (Liang et al. 2023)and Crohn’s Disease (Kostianovsky et al. 2023). A five-fold decrease in mRNA for MS4A2 (FCER1B, the beta subunit of the high-affinity IgE receptor) was also observed. This may suggest involvement of IgE-mediated mechanisms in CHS, because elevated IgE can induce a compensatory decrease in MS4A2/FCER1B expression (Brenzovich et al. 2009). The FCER1A IgE receptor had a 4-fold higher base mean expression (164.2) relative to FCER1B and showed a 3-fold down regulation in the sCHS patients. This could indicate that an IgE-mediated mechanism, such as an allergic reaction, is involved in CHS.

### HLA RNA expression levels

**Fig. 3:**
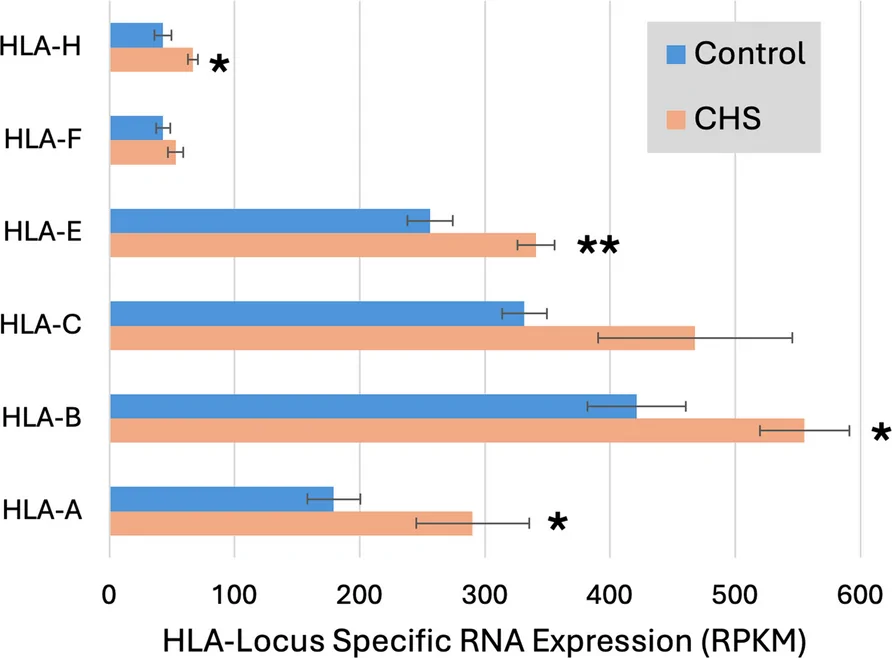
HLA-Locus Specific RNA Expression in suspected CHS Patients. RNAseq paired-end reads were realigned to an HLA-specific library using Bowtie2 and Seq2HLA. Average expression of MHC Class I isotypes is shown as RPKM. Error bars show SEM; * indicates *p* < 0.05 between groups

The observed upregulation of HLA-B and HLA-E, and downregulation of IgE receptor, in sCHS suggested a role for acquired immunity, and prompted detailed HLA expression and isotype analysis. RNAseq reads were realigned to a curated database using Seq2HLA. The QC metrics were consistent with the HISAT2 HG38 alignment. Overall read numbers did not differ significantly between groups (sCHS = 17.2 M, CON = 16.8 M, *p* = 0.75). However, reads aligning to the HLA region were significantly higher in sCHS (sCHS = 25,568, CON = 16,417, *p* = 0.007; Fig. 3). Differences in expression level were confined to HLA Class I: HLA-A (nRC sCHS = 290.1, CON = 179.2, *p* = 0.046), HLA-B (sCHS = 555.4, CON = 421.1, *p* = 0.026), and non-classical HLA-E (sCHS = 340.8, CON = 256.2,* p* = 0.004) and HLA-H (sCHS = 66.7, CON = 42.7, *p* = 0.012). Other HLA-I and all HLA-II alleles were not significantly different in expression level of their respective RNA transcripts (all *p* > 0.2).

### HLA digital isotyping

Seq2HLA analysis identified isotype-specific alleles with high confidence (*p* < 0.05) for almost all isotypes. All sCHS patients (7/7) carried at least one HLA-B allele previously associated with allergic hypersensitivity. Notably, one sCHS patient had the HLA-B27 allele, seen in 95% of ankylosing spondylitis cases, but only 5% of the general population (Supplementary Data 4). HLA-A and HLA-C alleles showed no systematic differences in isotypes (Supplementary Data 4).

**Fig. 4:**
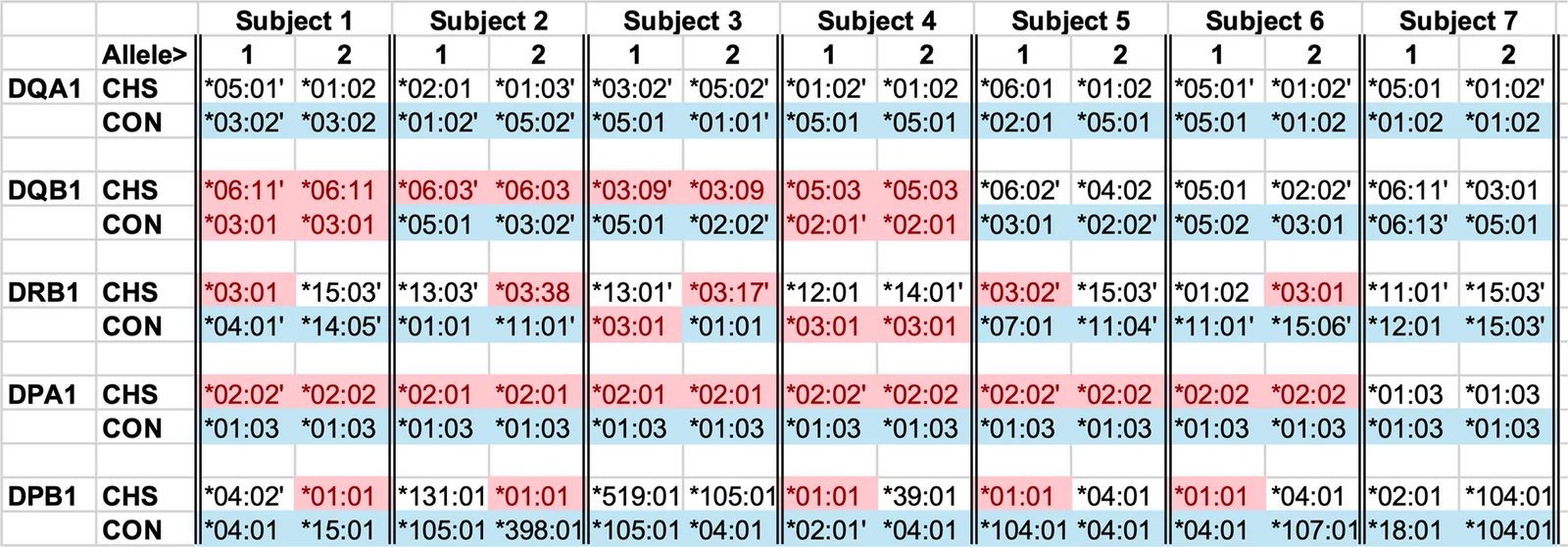
MHC Class II digital isotyping from RNAseq. RNAseq data was realigned and counted against an HLA-specific database as in Fig. 4. The aligned reads were used to calculate the major MHC Class II isotypes for each sCHS patient or Control (blue shade). Notable trends in the isotypes are highlighted in red. The DRA locus could not be reliably genotyped and the results are not shown

HLA Class II digital isotype analysis revealed three main findings (Fig. 4): (1) sCHS patients were more likely to be homozygous for DQB1 (sCHS 4/7 vs CON 2/7); (2) the DRB1*03 allele was more common in sCHS (sCHS 5/7, CON 2/7); and (3) nearly all sCHS patients carried DPA1*02 (sCHS 6/7 vs CON 0/7), an allele overrepresented compared to the expected frequency in Blacks (85.7% observed vs 23% expected, chi-square p = 0.007). Most DPA1*02 carriers were homozygous to four digits, while the one patient with DPA1*01:03 (used by all controls) also had the high-risk HLA-B27 allele. DPA1 allele calls had high read depth and confidence. A caveat is that from RNAseq isotyping it is difficult to differentiate between true homozygosity versus preferential allele usage in blood cells. Although limited by small sample size, these findings suggest that people with specific inherited HLA alleles may be prone to CHS, and warrant further study of HLA usage in CHS.

### Innate immune activation transcripts

Suspected CHS patients exhibited significant upregulation of innate immunity transcripts—particularly those related to neutrophil and monocyte activation. DEFA1 (neutrophil defensin A1) is a known biomarker of host response to bacterial infection (Chawla et al. 2016; Meltzer et al. 2023). Transcripts for INHBB, LTF, MMP9, and IL1R2 are neutrophil-associated and likely signal neutrophil activation. In the case of IFI27, it is a well-known signal of viral infection (Wargodsky et al. 2022; Min et al. 2020; Tang et al. 2017; Villamayor et al. 2023).

To validate the RNAseq findings, a panel of infection-related RNA biomarkers was analyzed by ddPCR. The ddPCR panel, previously validated in infection studies (Chawla et al. 2016; Meltzer et al. 2023; Wargodsky et al. 2022), confirmed increased RNA levels of DEFA1 (~ 2-fold), which is responsive to bacterial infection, and IFI27 (~ 3-fold), which is responsive to viral infection. This is consistent with the RNAseq data, though the ddPCR changes were of smaller magnitude than the RNAseq changes. Increases in IL8RB and ALPL, both responsive to bacterial infections, were also observed, but not RSAD2, which is responsive to viral infection, and none of the differences reached statistical significance (Supplementary Data 5). When compared to established thresholds for intra-abdominal infections, only one sCHS patient had an abnormal DEFA1 level (16.1% vs 10% threshold), and two patients had elevated scores for a biofilm-type infection (ALPL + IL8RB = 19.8% and 19.6%, vs 18% threshold). No patients had positive viral scores, and none of the Controls had abnormal results. Thus, although infection biomarkers were modestly elevated in some cases, most sCHS patients lacked a host immune response profile indicative of infection. However, it is likely that secondary infections resulting from hyperemesis in some patients are a contributing factor to the innate immune signal seen in the sCHS patients.

### Circulating bacterial/fungal DNA

Using methods developed for bacteremia in sepsis, total DNA was isolated from Tempus-preserved blood and analyzed by ddPCR for bacterial (Gram + and Gram– 16S) and fungal (23S) DNA. In contrast to septic patients in prior studies, sCHS patients showed no detectable increase in circulating pathogen DNA, indicating the absence of overt bacteremia despite the immune activation signature (not shown).

### Eicosanoid-related changes

A notable switch was observed in ALOX15 (down 10x) and ALOX15B (up 10x) expression in sCHS patients. While these are separate genes, their opposing regulation may reflect coordinated activity with potential implications for the ECS, as both are involved in arachidonic acid metabolism and can influence cannabinoid signaling. ALOX members are able to hydrolyze, and thus inactivate endogenous cannabinoids such as anandamide (AEA) and 2-AG (Ivanov et al. 2021). Changes in ALOX15 and ALOX15B may also relate to reticulocyte maturation, but no evidence of increased reticulocyte counts was found in sCHS patients.

### Adhesion/tight junction changes

Numerous DEGs were related to adhesion and tight junctions, processes integral to both immune cell communication and gut barrier integrity. A 4.4-fold decrease in ARPIN, a key tight junction component, was observed (Table 3). Reduced ARPIN is a hallmark of acute inflammation and has been identified as differentially methylated in heavy cannabis users (Chánez-Paredes et al. 2021; Fang et al. 2024). Other notable changes included decreased transcripts for AJAP1, EPHA2, PARVA, and protocadherins (PCDH18, PCDHGB), as well as increased IL-4, which may compromise intestinal barrier function (Colgan et al. 1994). Upregulation of metalloproteases (MMP8, MMP9) and proteases (ADAMTS2, hepsin, HTRA1) suggests increased extracellular matrix turnover and potential intestinal barrier disruption, paralleling findings in IBS (Hernández et al. 2023; O 2014). The striking increase in ADAMTS2 (> 100-fold) is particularly interesting because increases in the close family member, ADAMTS1, has been associated with hyperemesis of pregnancy (Timur and Guney 2022).

### Neurotransmission-related

Several transcripts implicated in brain-gut neurotransmission were altered in sCHS. Dopamine beta-hydroxylase (DBH) was decreased 5-fold (Table 2), consistent with changes seen in a post-operative nausea/vomiting (PONV) model (Sugino et al. 2021) and associated with migraine in GWAS. Chronic cannabis use is known to impact DBH activity (Markianos and Stefanis 1982). Conversely, monoamine oxidase A (MAOA), which degrades serotonin/5-HT, is increased 5-fold in the sCHS patients. Serotonin is a major regulator of the brain-gut axis and mediates both allergic and visceral hypersensitivity responses (Singh et al. 2023; Richards 2017). However, at present, it remains possible that these changes are a consequence of hyperemesis or haloperidol treatment, and thus, future controls will be important.

## Discussion

### Pharmacological hyperactivation of the ECS

Analysis of currently known transcripts within the endocannabinoid system (ECS) revealed no striking changes in cannabinoid receptors or related transcripts, but there were potentially important changes in the eicosanoid pathway, especially ALOX15, that directly impacts on the metabolism of endogenous cannabinoids. It remains possible that important alterations in ECS signaling are not reflected at the mRNA level, warranting further investigation using alternative approaches. Further, we cannot exclude changes in ECS signaling that might occur in the brain regions that regulate emesis. CHS users often use hot showers to mitigate symptoms, which has been suggested as evidence for heat-induced changes in TRPV1, a known ECS modulator (Loganathan et al. 2024).

### Cannabis-induced epigenetic changes in gene/protein expression

Previous evidence for altered methylation of genes such as ARPIN is strengthened by the present findings of reduced RNA expression for these transcripts in sCHS. While it is possible this is coincidental, it is interesting to speculate that long-term, high-dose exposure to cannabinoids induces epigenetic changes—including DNA methylation and histone modification—which impact gene and protein expression in CHS.

### Gut barrier function alterations

The observed increase in proteolytic enzyme transcripts with concomitant reductions in protease inhibitors and key adhesion proteins, particularly ARPIN, may reflect a progressive loss of gut barrier function, assuming that these changes are paralleled in the GI tract. Notably, similar imbalances between proteolytic and anti-proteolytic pathways have been proposed as contributing to the ‘soft collagen’ theory of IBS, where genetic variants in connective tissue genes combined with infection or inflammation drive pronounced GI inflammation (Vounotrypidis and Kouklakis 2010). In CHS, a comparable imbalance may disrupt the GI barrier, causing leakage of bacteria and bacterial products, thus triggering pain and emesis. While we could not detect elevated bacterial DNA in blood, future studies could emply more sensitive measures of gut barrier function. Communication between the gut epithelial barrier and immune cells is essential to maintaining barrier integrity (Yao et al. 2024). Cannabis particulates would reach the GI tract by swallowing particulates that stay in the mouth, sinuses, and throat, and particulates that reach the lungs, but are moved back to the throat by mucociliary clearance and then swallowed (Crawford et al. 2021). For instance, cigarette smoke and environmental pollutants are known contributors to intestinal barrier dysfunction and GI inflammation in Crohn’s and ulcerative colitis (Crawford et al. 2021; Berkowitz et al. 2018). There are large epidemiological studies involving roughly 10,000 patients each that observed that edible cannabis products are associated with a higher (Glodosky et al. 2026)or lower (Monte et al. 2019) incidence of CHS, and so it is difficult to determine how elevated gut levels of cannabis would affect CHS development.

Loss of mucosal integrity would permit the gut microbiome and its soluble products to interact with immune cells in the submucosa. In particular, gut enterochromaffin cells are key drivers of visceral pain and anxiety (Bayrer et al. 2023). Histamine released from mast cells in response to IgE activates these enterochromaffin cells (Pfanzagl et al. 2019), which are the primary source of serotonin (5-hydroxytryptamine, 5-HT)—a major neurotransmitter within the central nervous system (CNS) and GI tract that regulates peristalsis and can trigger hyperemesis (Grundy 2008; Crowell et al. 2004). Notably, changes in transcripts such as NECAB2 (Zhang et al. 2018)and GPER1 (Jouffre et al. 2023; Xu et al. 2020) have been linked to visceral hypersensitivity and pain.

### Immune-related pathways

**Fig. 5:**
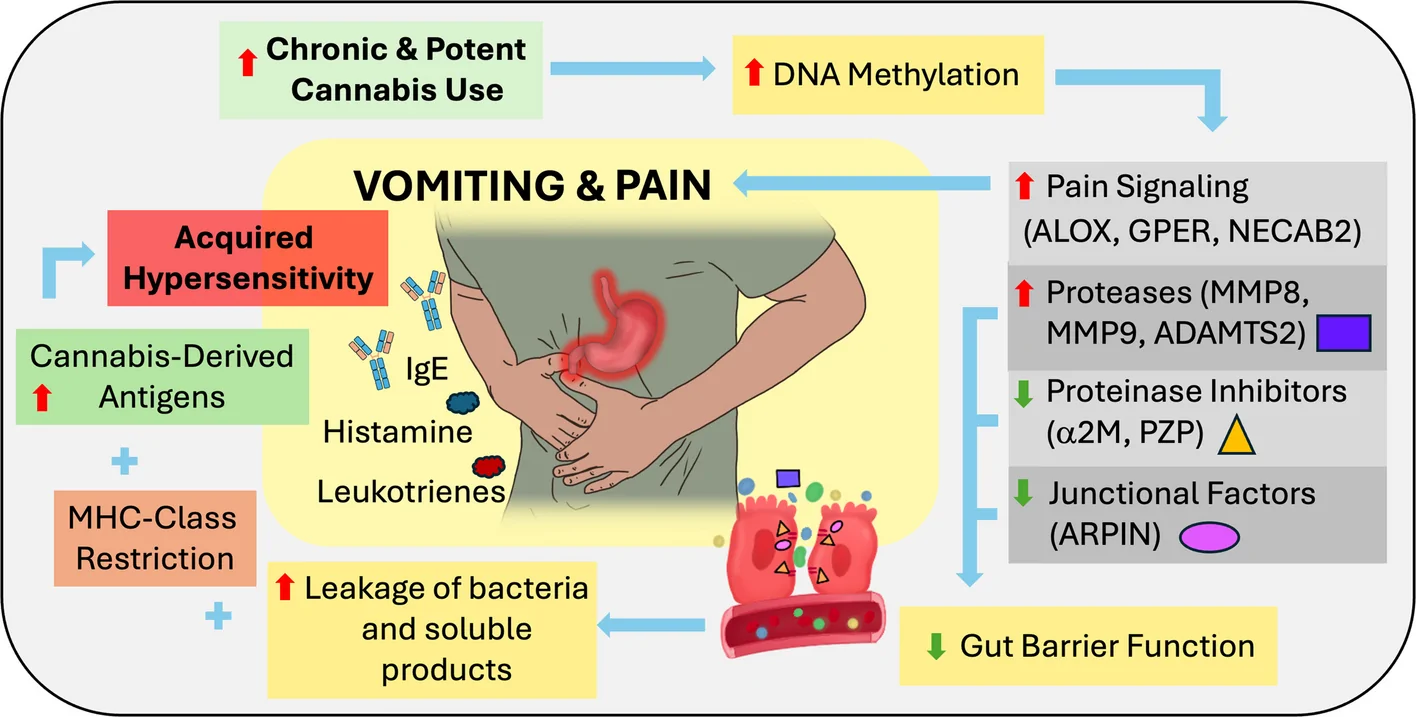
Graphic summary of transcriptome changes in suspected CHS. The preliminary genome-wide transcriptomic analysis in sCHS suggests changes in several potentially related systems. There is prior evidence that heavy use of high-potency cannabis could induce stable changes in gene expression via epigenetic modifications such as DNA methylation, affecting both junctional/adhesive and proteolytic/anti-protease pathways. The result may be alterations in transcripts that maintain gut barrier integrity, permitting leakage of microbiome and cannabis products into the submucosa. The mucosal immune response may then be dysregulated, producing a hypersensitivity reaction to cannabis components, especially in HLA-restricted subjects

A number of the transcript changes observed could relate to an altered immune response to one or more cannabis components (Fig. 5). Multiple lines of evidence support this: (1) B cell activation; (2) T cell suppression; (3) neutrophil activation; (4) HLA restriction and increased expression; (5) IgE receptor down-regulation; (6) lipoxygenase imbalance (ALOX15/15B); and (7) changes in interleukins and their receptors. It is conceivable that pairing cannabis byproducts in the gut with a concurrent viral or bacterial infection may sensitize patients to cannabis antigens, similar to the development of α-gal syndrome following tick exposure. Certain cannabis antigens, such as the non-specific lipid transfer protein (nsLTP), are known to provoke allergic and even anaphylactic reactions in susceptible individuals (Ebo et al. 2024). Reports of “cannabis allergy” have increased in parallel with CHS (Decuyper et al. 2015). Thus, it is interesting to speculate that CHS may involve an adaptive immune response—possibly involving co-exposure to cannabis and classical pathogenic antigens. This could create a hypersensitivity reaction to cannabis components, activating gut-innervating sensory neurons and resulting in a hyperemesis syndrome (Wallrapp and Chiu 2024). The restriction of the reaction to the gut could be due to some necessary processing of the cannabis allergen by gut enzymes.

Gastrointestinal disorders and food allergies often show HLA associations. For example, in celiac disease, unknown environmental triggers elicit adverse reactions to gluten (Crawford et al. 2021), with both celiac disease and IBS strongly associated with HLA-DQ2/DQ8 (Berkowitz et al. 2018; Glodosky et al. 2026; Bayrer et al. 2023). HLA-B52 and HLA-DR15 have been linked to early-onset ulcerative colitis in Japanese children (Pfanzagl et al. 2019). In animal models of HLA-B27 spondylarthritis, compromised gut barriers allow microbial antigens to translocate and trigger immune responses (Grundy 2008). GWAS studies in food allergy consistently highlight genes involved in barrier and immune function—including HLA and IL-4 (Crowell et al. 2004; Zhang et al. 2018). Epigenome-wide association studies also repeatedly identify IL-4 among the top candidates for food allergy (Jouffre et al. 2023). Food protein-induced enterocolitis syndrome (FPIES), a non-IgE food allergy, can affect up to 1% of children and 0.22% of adults, presenting with severe vomiting episodes reminiscent of CHS (Xu et al. 2020). Surprisingly, there are very limited studies measuring immune changes in CHS, which have described elevated white blood cell counts with associated neutrophilia (75.8%) and mild hypokalemia (57.9%). Lipase is typically not elevated, and C-reactive protein remains less than 50 mmol/L in most cases (98.2%) (Ebo et al. 2024).

## Conclusions

- There were not notable differences in the RNA levels of the major ECS components in sCHS patients, although changes in eicosanoid enzymes could impact endogenous cannabinoids.
- However, there were striking differences in the RNA expression levels of B cell immunoglobin genes, as well as higher expression of HLA Class I genes, and potential restriction of the HLA Class II allele usage in the sCHS patients.
- There was evidence of host immune activation of neutrophil biomarkers of infection, but this could be easily explained by complications secondary to the hyperemesis.
- The pattern of changes suggests that future studies should include a careful analysis of adaptive immune functions and usage of inherited HLA alleles in CHS patients.

### Limitations

This pilot study is limited by its small sample size and demographic differences between suspected CHS patients and controls, which may confound transcriptomic and HLA findings. Ideally, this study would have used prospectively diagnosed CHS and longitudinal abstinence-confirmed follow-up, as well as tracking potentially relevant co-morbid conditions such as migraine, atopic disease, food allergy, celiac disorders and Crohn’s/IBS. The absence of control groups with emesis unrelated to cannabis use, or chronic cannabis users without CHS, restricts our ability to isolate CHS-specific effects from those of emesis or cannabis exposure alone. However, in prior studies of patients with abdominal pain unrelated to CHS, changes in neutrophil activity, such as DEFA1, were observed, but changes in the adaptive immune and HLA pathways were not, suggesting that the present results are probably not generic responses to abdominal pain (Chawla et al. 2016). Additionally, as our data are derived from whole blood rather than gut tissue, the extent to which these biomarkers reflect changes in the brain/gut axis is uncertain.

## Future directions

Future studies should investigate more deeply into the potential immune mechanisms underlying CHS. While total IgE may be nonspecific, assays for cannabis-specific IgE could be highly informative. Expanding HLA genotyping into larger, more diverse CHS cohorts will help determine the extent of genetic susceptibility and HLA restriction. The inclusion of additional control groups—such as chronic cannabis users without emesis and patients with emesis unrelated to cannabis—will be essential to distinguish the specific contributions of cannabinoids and emesis to the CHS transcriptomic profile. Finally, investigating familial aggregation, sibling or twin concordance, and the heritability of CHS could provide critical insight into the genetic and environmental interplay underlying disease susceptibility, particularly in relation to HLA genotype and immune sensitization.

## Supplementary Information

Supplementary Material 1.

Supplementary Material 2.

Supplementary Material 3.

Supplementary Material 4.

Supplementary Material 5.

## Data Availability

The full RNAseq dataset, as normalized and raw read counts, is available at NCBI Gene Expression Omnibus (GEO) Accession GSE303922. The raw sequence data (fastq) files contain personally identifiable data and therefore can be shared only with appropriate protections for the subjects. The digital PCR RNA biomarker panel for host immune activation can be obtained from True Bearing Diagnostics, Inc. by purchase or license agreement.

## Abbreviations

CBC: Complete blood count
CHS: Cannabinoid hyperemesis syndrome
ddPCR: Droplet digital PCR
DEGs: Differentially expressed genes
DND: Daily or near daily
ECS: Endocannabinoid system
ED: Emergency department
IBS: Irritable bowel syndrome
HLA: Human leucocyte antigen
MHC: Major histocompatibility complex
nRC: Normalized read count
RIN: RNA integrity number
RNAseq: RNA sequencing
rRNA: Ribosomal RNA
