# Supplementary material for: Whole-blood transcriptomic findings in emergency department patients with suspected cannabinoid hyperemesis syndrome: a pilot study
Source: J Cannabis Res. 2026 Jul 23;8:110. doi: 10.1186/s42238-026-00477-x (PMC13685215; doi:10.1186/s42238-026-00477-x)
Supplement: Supplementary file 1 [file 42238_2026_477_MOESM3_ESM.docx]

Supplementary Table 3. ECS Pathway
